# Stem Cells from Human Exfoliated Deciduous Teeth Ameliorate Autistic-Like Behaviors of *SHANK3* Mutant Beagle Dogs

**DOI:** 10.1093/stcltm/szac028

**Published:** 2022-05-24

**Authors:** Lu Zhao, Yuan Li, Xiaoxing Kou, Benchi Chen, Jing Cao, Jun Li, Jianqi Zhang, Heng Wang, Jianping Zhao, Songtao Shi

**Affiliations:** Hospital of Stomatology, Guanghua School of Stomatology, Sun Yat-sen University, South China Center of Craniofacial Stem Cell Research, Guangdong Provincial Key Laboratory of Stomatology, Guangzhou, People’s Republic of China; Beijing Sinogene Biotechnology Co. Ltd, Changping District, Beijing, People’s Republic of China; Hospital of Stomatology, Guanghua School of Stomatology, Sun Yat-sen University, South China Center of Craniofacial Stem Cell Research, Guangdong Provincial Key Laboratory of Stomatology, Guangzhou, People’s Republic of China; Key Laboratory of Stem Cells and Tissue Engineering (Sun Yat-sen University), Ministry of Education, Guangzhou, People’s Republic of China; Beijing Sinogene Biotechnology Co. Ltd, Changping District, Beijing, People’s Republic of China; CAR-T (Shanghai) Biotechnology Co. Ltd, Yangpu District, Shanghai, People’s Republic of China; Key Laboratory of Stem Cells and Tissue Engineering (Sun Yat-sen University), Ministry of Education, Guangzhou, People’s Republic of China; Beijing Sinogene Biotechnology Co. Ltd, Changping District, Beijing, People’s Republic of China; Beijing Sinogene Biotechnology Co. Ltd, Changping District, Beijing, People’s Republic of China; Beijing Sinogene Biotechnology Co. Ltd, Changping District, Beijing, People’s Republic of China; Hospital of Stomatology, Guanghua School of Stomatology, Sun Yat-sen University, South China Center of Craniofacial Stem Cell Research, Guangdong Provincial Key Laboratory of Stomatology, Guangzhou, People’s Republic of China; Key Laboratory of Stem Cells and Tissue Engineering (Sun Yat-sen University), Ministry of Education, Guangzhou, People’s Republic of China; International Center for Aging and Cancer, Hainan Medical University, Haikou, People’s Republic of China

**Keywords:** stem cells from human exfoliated deciduous teeth, mesenchymal stem cells, autism, *SHANK3*, interferon-γ, interleukin-10

## Abstract

Mesenchymal stem cell-based therapy has emerged as a great potential approach to treat individuals with autism spectrum disorders (ASD), a group of developmental disabilities characterized by impairments in social interaction and communication. Stem cells from human exfoliated deciduous teeth (SHED), holding earlier developing characteristics, have immune-modulatory and anti-inflammatory properties. To investigate whether SHED transplantation can rescue autistic-like symptoms in *SHANK3* mutant beagle dogs, 12 *SHANK3* mutant beagle dogs were randomly assigned into 2 groups according to their behavior evaluated by social interaction tests. Six mutant dogs received 6 intravenous infusions of SHED and were followed up for 3 months by testing social interaction and inflammatory cytokine levels. We found that infusion of SHED significantly improved impaired social novel preference of *SHANK3* mutant beagle dogs at 1- and 3-month follow-ups. Social intimacies (following, sniffing, and licking) between mutant beagle dogs and human experimenters were partly improved. Stressed tail posture, indicating social stress, was also significantly alleviated. In addition, we showed that the levels of serum interferon-γ and interleukin-10 were notably increased and decreased, respectively, in *SHANK3* mutant beagle dogs. Infusion of SHED was able to rescue altered interferon-γ and interleukin-10 levels. We failed to observe any serious adverse events after infusion of SHED. In summary, SHED transplantation may be a safe and effective therapy for ASD. The correction in the levels of serum interferon-γ and interleukin-10 may serve as an index to predict autistic severity and therapeutic outcomes.

## Graphical Abstract

**Graphical Abstract:**
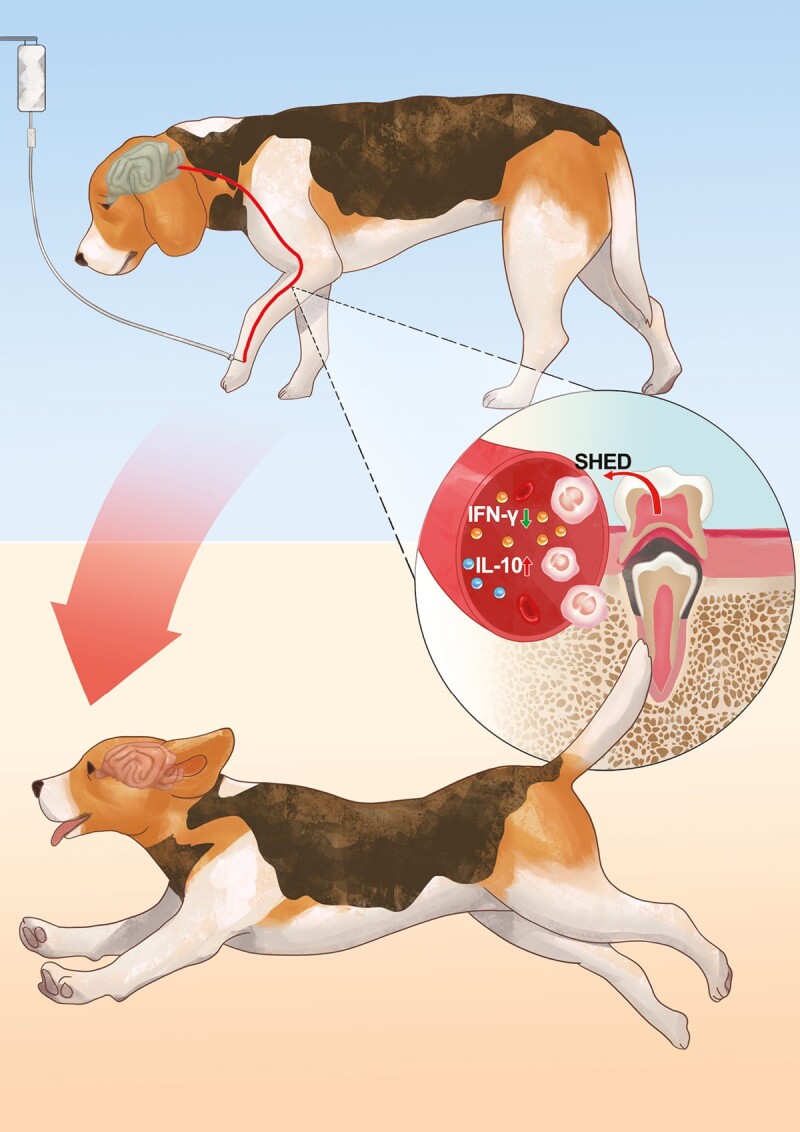

**Significance Statement**

Intravenous stem cells from human exfoliated deciduous teeth (SHED) transplantation can effectively alleviate the autistic-like symptoms of impaired social novelty preference and social stress in *SHANK3* mutant beagle dogs. The levels of serum interferon-γ and interleukin-10 were notably increased and decreased, respectively, in *SHANK3* mutant beagle dogs. Infusion of SHED can rescue altered interferon-γ and interleukin-10 levels. Our study confirmed that the *SHANK3* mutant beagle dog model may be valuable for evaluating SHED-mediated therapy in autism. Stem cells from human exfoliated deciduous teeth transplantation may serve as a simple, safe, and effective therapy for autism.

## Introduction

Autism spectrum disorders (ASD) are a group of complex developmental disorders associated with deficits in social interaction, restricted interests, and/or stereotyped behaviors.^1^ Additionally, gastrointestinal problems, sleep disorders, attention deficits, and intellectual disability are very common in children with ASD.^2^ The prevalence of ASD was approximately 2% in 8-year-old children, with 4 times as many boys affected than girls.^3^ For the treatment of ASD, multiple approaches have been applied, including intensive behavioral therapy, occupational therapy, speech therapy, and psychotropic medications.^4^ However, there is no approved medicine that can ameliorate autistic core symptoms.

The etiology of ASD is not fully understood. It may involve in a complex combination of genetic and environmental factors.^5^ In recent years, accumulated evidence showed that immune dysfunction and neuroinflammation may be involved in the pathophysiology of ASD.^6-8^ Thus, immunomodulatory therapy may hold promise for treating ASD. Mesenchymal stromal cells (MSCs) with immune-modulatory capacities have been isolated from different tissues. Previous reports showed the safety and a certain degree of efficacy of MSC treatment for patients with ASD.^9,10^ The exact mechanism of MSC therapy and its long-term therapeutic effect are largely unknown.

Stem cells from human exfoliated deciduous teeth (SHED) are derived from neural crest cells and possess an elevated proliferation rate, number of population doublings, osteo-differentiation capacity, and immune-modulatory capacities compared with bone marrow MSCs.^11^ Importantly, SHED, derived from the deciduous tooth, may represent an early stage of stem cell population when compared with adult tooth-derived dental pulp stem cells. They express a variety of neural cell markers including nestin, βIII-tubulin, NeuN, glial fibrillary acidic protein, and neurofilament medium polypeptide protein.^11-14^ When injected into the hippocampus of the mouse brain, SHED survive inside the brain and express neural markers.^11^ Stem cells from human exfoliated deciduous teeth have been used to treat a variety of neuroinflammation-related diseases, including spinal cord injury, hypoxic-ischemic brain injury, and experimental autoimmune encephalomyelitis through their neuro-regenerative, anti-inflammatory, and immunomodulatory activities.^12,15,16^ Because SHED can be derived in a non-invasive manner from exfoliated deciduous teeth, they are easily accessed as naturally disposed organs with limited ethical or legal concerns. Stem cells from human exfoliated deciduous teeth thus have multiple advantages in clinical use for neural diseases such as ASD.

Autism spectrum disorders animal models can help to understand ASD-related pathogenesis and develop therapeutic strategies. Mutations in *SHANK3* have been characterized in drosophila, zebrafish, mouse, rat, and monkey.^17,18^ Previous studies have provided insights into the mechanism underlying *SHANK3*-associated ASD.^17,19,20^ In this study, we used CRISPR/Cas9-mediated heterozygous *SHANK3* mutation beagle dogs as a model to explore the safety and efficiency of SHED therapy for ASD.

## Materials and Methods

### Animal

Male beagle dogs of 5 to 7 months old with CRISPR/Cas9-mediated heterozygous *SHANK3* mutation and age-matched wild-type male beagle dogs (Beijing Sinogene Biotechnology Co. Ltd., Beijing, China) were housed and tested for their behaviors at Beijing Sinogene Biotechnology Co. Ltd. All procedures were performed according to the Guidelines of the Institutional Animal Care and Use Committee of the Beijing Sinogene Biotechnology Co. Ltd. (XNG-IAC-20191201).

### Stem Cells from Human Exfoliated Deciduous Teeth Preparation

Ethics approval for collection and use of SHED was approved by the Medical Ethics Committee of Hospital of Stomatology, Sun Yat-sen University (project ID: KQEC-2021-48-01). SHED were isolated and cultured as reported previously.^21^ Briefly, 25 normal humans exfoliated deciduous teeth were collected from 20 different donors (6-12 years old) as a stem cell resource pool. The dental pulp was separated and then digested in a solution of 3 mg/mL collagenase type I (Worthington Biochemical Corporation, Lakewood, USA) and 4 mg/mL dispase (Roche Diagnostics GmbH, Mannheim, Germany) for 1 h at 37 °C. Single-cell suspensions were cultured and the cells were expanded to 90% confluence (10-14 days) to establish the passage (P) 0 culture. For passaging, cells were digested with TrypLE (Gibco, Thermo Fisher Scientific, Waltham, USA) and expanded to P3 using α-MEM (Gibco, Thermo Fisher Scientific) containing 15% fetal bovine serum (Gibco, Thermo Fisher Scientific), 2 mM l-glutamine (Gibco, Thermo Fisher Scientific), 100 U/mL penicillin and 100 μg/mL streptomycin (Invitrogen, Waltham, USA), and 10 mM l-ascorbic acid phosphate (Wako, Tokyo, Japan). were characterized by cell morphology, surface markers, and multi-differentiation potential according to our previous study (Supplementary Fig. S1A-E).^11^ Stem cells from human exfoliated deciduous teeth from this stem cell pool were suspended in 100 mL saline solution and infused into *SHANK3* mutant beagle dogs intravenously. The total cell number was calculated as 3 × 10^6^ cells per kg body weight (Supplementary Fig. S1F for baseline characteristics of SHED transplantation units).

### Study Design

**Figure 1.**
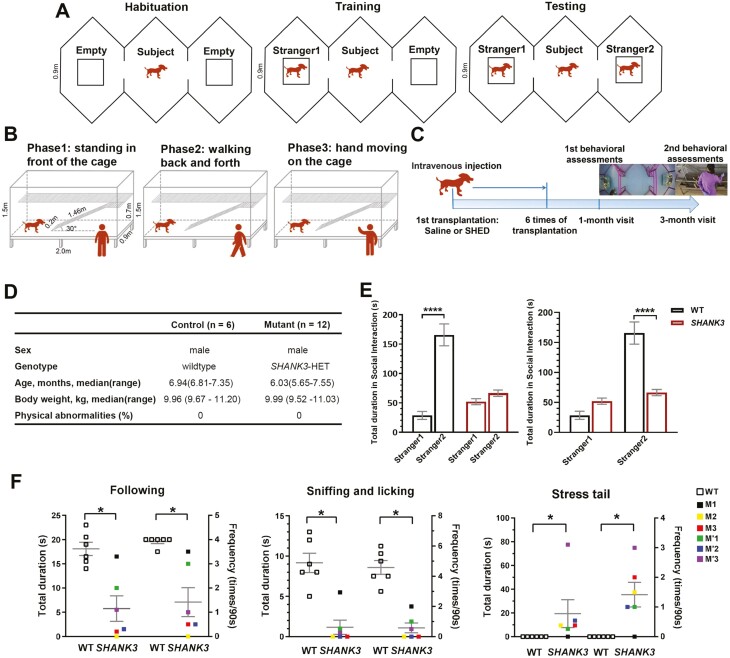
Impaired social novelty preference (SNP) and social interaction in *SHANK3* mutant beagle dogs. (**A**) Schematic diagram illustrating the 3-chamber sociability test. (**B**) Schematic diagram illustrating the dog-and-experimenter interaction test. (**C**) A scheme presenting the experimental design. After 6 intravenous SHED infusions in SHANK3 mutant beagle dogs at age of 5-7 months, behavior changes were evaluated by the 3-chamber test and dog-and-experimenter interaction test after 1 month and 3 months following SHED treatment. (**D**) Baseline characteristics of wild-type (WT) and *SHANK3* mutant beagle dogs. (**E**) Impaired SNP in *SHANK3* mutant beagle dogs. Wild-type dogs spent longer duration of interactions in the chamber containing the Stranger2 than in the chamber containing Stranger1, displaying the preference for social novelty. *SHANK3* mutant dogs showed no significant differences in the total duration of interactions with the Stranger1 and Stranger2. Moreover, *SHANK3* mutant dogs spent less time interacting with Stranger2 compared with WT dogs (*n* = 6 in WT, *n* = 12 in *SHANK3*). (**F**) Reduced social interaction and increased social stress in *SHANK3* mutant beagle dogs. Compared with WT dogs, *SHANK3* mutant dogs showed significantly decreased frequency and duration of following, sniffing, and licking, while significantly increased stress-tail frequency and duration compared with WT dogs (*n* = 6 in WT, *n* = 6 in *SHANK3* named M1, M2, M3, M’1, M’2, and M’3). Data are presented as mean ± SEM. *P < .05, ****P < .0001.

In our study, 12 *SHANK3* mutant beagle dogs were equally grouped into untreated (*SHANK3* + saline group) and SHED-treated groups (*SHANK3* + SHED group) with 6 mutant dogs per group according to their behavior under developmental impairment conditions evaluated by the 3-chamber test (Fig. 1A) and social interaction test with experimenters (Fig. 1B). Specific testing methods and results were noted below. Six age-matched wild-type beagle dogs were a control group. A scheme presented the experimental design (Fig. 1C). Mutant beagle dogs in the *SHANK3* + SHED group received an infusion every 7-10 days for a total of 6 times. Wild-type beagle dogs and mutant dogs in the *SHANK3* + saline group were given equal volumes of saline infusion. All the beagle dogs were then behaviorally evaluated in 1-month and 3-month post-treatment. Complete blood count and blood biochemical examination of SHED-treated mutant dogs were performed before and after treatment. The details of the wild-type and mutant beagle dogs were shown in Fig. 1D.

### Behavioral Tests

#### Three-Chamber Test of Social Novelty Preference

We used a 3-chamber test to examine the social novelty preference (SNP) of beagle dogs (Fig. 1A).^22,23^ Each chamber was a hexagon of 0.9 m per side. For habituation, the subject dog was allowed to familiarize itself with the chambers and environment freely for 10 minutes. Then, an unfamiliar dog (Stranger1) in a cage was placed in one side-chamber, while another empty cage was placed in the other side-chamber. The subject dog was allowed to explore the 3 chambers freely for 10 minutes. In the test, a second stranger dog (Stranger2) was then placed in the empty cage and the subject dog was again allowed to explore all 3 chambers freely for 10 minutes. The position of Stranger1 and 2 within the 3-chamber was random between tests. All stranger dogs were the same age and sex as the subject dog. The behaviors of the subject dogs were recorded by a video camera (HST-T236U4RZE, Hiside, Shenzhen, China), and the duration of different social behaviors were analyzed manually by trained experimenters who were blind to the genotypes and experimental groups of the subject dogs.

#### Social Interaction Test Between Dogs and Experimenters

To examine the social interaction between home-caged dogs and experimenters, we designed a test based on the classical behavioral test for beagle dogs (Fig. 1B).^24,25^ The test was performed once daily (90 s a session) for 2 consecutive days. The test includes 3 steps: Step 1, an experimenter who was familiar with the subject dogs stood in front of the home-cage for 30 s; Step 2, the experimenter walked back and forth twice in front of the home-cage for 30 s; Step 3, the experimenter placed their hands on the cage and moved their fingers for 30 s. A video camera (DS-IPC-T12H2-I, Hikvision, China) was placed in front of the home-cage to record the behaviors of the dog during the 3-step 90 s assay. The duration and frequency of each specific behavior including following the experimenter in Step 2, sniffing or licking the experimenter’s hands in Step 3, and stressed tail positioning (stress tail) were statistically analyzed during the 90 s assay. Stress tail was defined as the tail being held stiff and low or tucked between the hind limbs, signaling fear, anxiety, or nervousness.^22^

### Serum Inflammatory Cytokine Evaluation

Blood was collected from each of the beagle dogs 1 day before SHED treatment as well as 1 and 3 months after the treatment and allowed to clot at room temperature for 60 minutes. Clotted samples were centrifuged at 3000 rpm for 20 minutes, and serum was removed, aliquoted, and stored at −80 °C until use. Levels of serum inflammatory cytokines were analyzed using commercially available cytokine ELISA kits (Quantikine ELISA Kits, R&D Systems, USA; Maisha Industries, China).

### Statistical Analysis

All results were analyzed using Microsoft Excel (Microsoft Office 365 package, San Diego, CA), Prism GraphPad 8.0 (Prism GraphPad Software, San Diego, CA), and SSPS for Windows software version 20.0 (SSPS Inc., Chicago, IL) programs and all data were represented in graphs as the mean ± standard error of the mean (SEM). All analyses were performed on a blinded basis. The normal distribution of the data was determined using Kolmogorov-Smirnov test. *P*-values were calculated using the Student’s *t* test between the means of 2 groups and the non-parametric Mann-Whitney test, whenever distributions were not normal. Analysis of variance (ANOVA) was also used to compare the results between 3 or more groups, followed by Tukey’s post hoc test. 2-way ANOVA was used to analyze the differences in social interaction. The correlation between the serum inflammatory cytokines and SNP was analyzed using Prism GraphPad 8.0 and Pearson’s test was applied for calculating correlations. The multiple linear regression analysis was performed with SPSS version 20.0. *P*-value summaries were as follows: *P < .05; **P < .01; ***P < .001; ****P < .0001.

## Results

### Impaired SNP and Social Interaction in *SHANK3* Mutant Beagle Dogs

A 3-chamber sociability test was used to determine SNP. According to the typical 3-chamber sociability test, Stranger1 was in one side-chamber as a familiar social stimulus, and Stranger2 was placed in the other side-chamber as a novel one.^23,25^ For the wild-type beagle dogs, they spent longer duration in the chamber containing Stranger2 than in the chamber containing Stranger1, showing a typical preference for interaction with the novel dog (Fig. 1E). However, *SHANK3* mutant beagle dogs showed the impaired SNP, as noted by no significant differences in the total duration of interactions with the Stranger1 and Stranger2 (Fig. 1E**).** Moreover, *SHANK3* mutant dogs spent less time interacting with Stranger2 compared with wild-type dogs (Fig. 1E).

We next examined social interaction by performing a dog-and-experimenter interaction test. Considering the individual differences of mutant beagle dogs and the fact that not all mutant dogs showed obvious deficits of social interaction at 5-7 months old, 6 mutant dogs were selected due to abnormal behaviors in the dog-and-experimenter interaction test. Specific results were as follows: *SHANK3* mutant dogs did not actively initiate or respond to social interaction with the experimenter, as shown by the significantly reduced frequency and shorter duration of following, sniffing, and licking compared with wildtype dogs (Fig. 1F). In addition, *SHANK3* mutant dogs displayed social stress with more frequent and longer duration of stress tail (Fig. 1F). Instead, *SHANK3* mutant dogs showed impaired SNP and social interaction, which parallel some typical aspects of autistic phenotypes in humans.^1^

Considering the reliability of SHED treatment outcomes, the 6 mutant dogs with deficits of social interaction were randomly and equally allocated into the *SHANK3* + saline group (*n* = 3, named M1, M2, M3) and the *SHANK3* + SHED group (*n* = 3, named M’1, M’2, M’3). The other 6 mutant dogs without obvious defects in social interaction were also equally distributed into the 2 groups (*SHANK3* + saline group, M4, M5, M6; *SHANK3* + SHED group, M’4, M’5, M’6).

### Blood Biochemistry Tests of *SHANK3* Mutant Beagle Dogs

During the period of SHED transplantation and follow-up visits, no allergic immune responses or other serious adverse events were observed in the SHED-treated group. In SHED-treated mutant beagle dogs, γ-glutamyl transpeptidase levels showed a significant increase, while the phosphorus, calcium, and alkaline phosphatase levels were significantly lower compared to the pre-treatment (Table 1). Despite changes in some indicators, there were no deviations outside of reference ranges in complete blood count/biochemical examination before and after treatment.

**Table 1.** Blood biochemistry tests of *SHANK3* mutant beagle dogs.

Parameter	Pre-treatment (mean ± SEM)	Post-treatment (mean ± SEM)	Reference range	Unit
RBC	7.15 ± 0.39	8.38 ± 0.67	5.5-8.5	10^12^/L
Hemoglobin	153.80 ± 7.81	166.20 ± 7.84	120-180	g/L
Hematocrit	50.78 ± 2.79	54.23 ± 2.40	37-55	FL
MCV	70.97 ± 0.29	70.83 ± 0.63	60-77	FL
MCH	21.55 ± 0.17	21.68 ± 0.21	19.5-24.5	Pg
MCHC	303.33 ± 2.59	306.33 ± 2.42	320-360	g/L
Platelet	3.79 ± 0.22	2.6 ± 0.95	2-9	×10^11^/L
WBC	14.67 ± 2.36	11.45 ± 0.82	6-17	×10^9^/L
Lymphocyte	17.40 ± 3.95	28.28 ± 3.72	12-30	%
CRP	8.17 ± 4.04	13.07 ± 3.34	0-9.9	μg/L
Glucose	5.48 ± 0.36	4.94 ± 0.31	4.11-7.95	mmol/L
Creatinine	58.33 ± 4.75	60.67 ± 2.73	44-159	μmol/L
Urea	6.02 ± 0.55	4.83 ± 0.27	2.5-9.6	mmol/L
Phosphorus	2.79 ± 0.074	1.89 ± 0.03*	0.81-2.20	mmol/L
Calcium	2.64 ± 0.035	2.25 ± 0.035*	1.98-3.00	mmol/L
Total protein	60.33 ± 2.22	60.50 ± 1.77	52-82	g/L
Albumin	28.67 ± 0.76	28.83 ± 0.79	23-40	g/L
Globulin	31.83 ± 1.64	31.67 ± 1.15	25-45	g/L
ALT	33.83 ± 6.81	39.17 ± 5.95	10-125	IU/L
ALKP	110.5 ± 5.61	90.17 ± 4.14*	23-212	IU/L
GGT	1.17 ± 0.40	3.67 ± 0.67*	0-11	IU/L
TBIL	<2	<2	0--15	μmol/L
CHOL	4.19 ± 0.17	3.87 ± 0.13	2.84--8.26	mmol/L
AMYL	512.17 ± 42.42	490.83 ± 21.50	500--1500	IU/L
LIPA	698.50 ± 128.66	731.83 ± 94.54	200--1800	IU/L
LDH	275.50 ± 23.25	324.50 ± 78.80	40--400	IU/L

Data are presented as mean ± SEM and analyzed using Student’s *t* test. *n* = 6,*P < .05.

Abbreviations are as follows: RBC, red blood cell; MCV, mean corpuscular volume; MCH, mean corpuscular hemoglobin; MCHC, mean corpuscular hemoglobin concentration; WBC, white blood cell; CRP, C-reactive protein; ALT, alanine transferase; ALKP, alkaline phosphatase; GGT, γ-glutamyl Transpeptadase; TBIL, total bilirubin; CHOL, cholesterol; AMYL, serum amylase; LIPA, pancrelipase; LDH, lactate dehydrogenase.

### Improved SNP and Reduced Social Stress in *SHANK3* Mutant Beagle Dogs Upon SHED Transplantation

**Figure 2.**
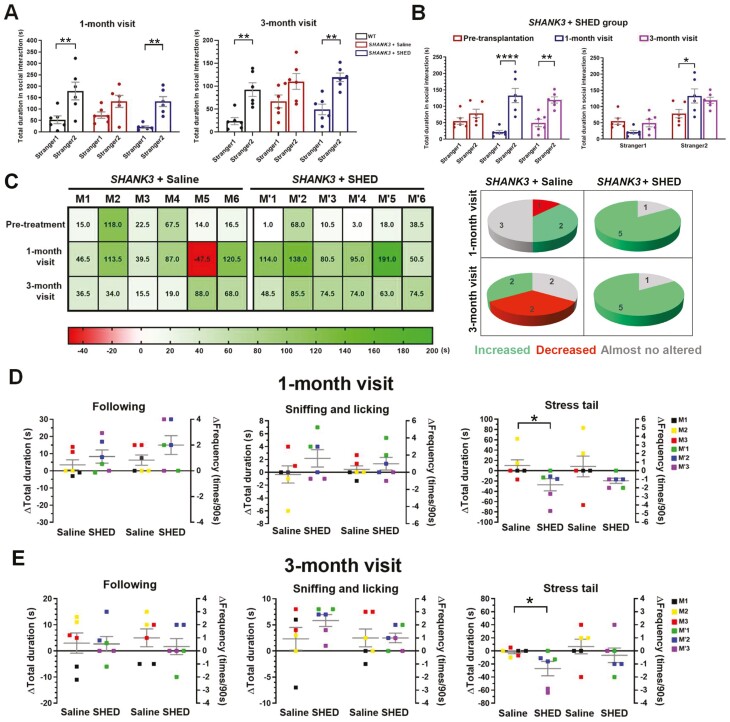
Effects of SHED treatment on SNP and social interaction in *SHANK3* mutant dogs. (**A**) In the 1-month and 3-month visits, *SHANK3* mutant dogs treated with SHED restored SNP with similar behavior as the WT, displaying significant differences in the duration of interactions between the Stranger2 and Stranger1, while untreated mutant dogs exhibited no significant differences between the 2 dogs (*n* = 6 per group). (**B**) Stem cells from human exfoliated deciduous teeth treatment markedly improved SNP in *SHANK3* mutant dogs as evidenced by increasing active social duration with Stranger2, especially in the 1-month visit (*n* = 6). (**C**) Individual SNP of each mutant dog was evaluated before treatment, 1-month post-treatment, and 3-month post-treatment to assess social time-difference (ST_dif_) between the Stranger2 and Stranger1. The heatmap was generated with green representing the positive ST_dif_ (Stranger2 > Stranger1) and red representing the negative ST_dif_ (Stranger2 < Stranger1). Pie charts summarized the heatmap results indicating the number of mutant dogs with increased, decreased, or almost no altered ST_dif_ (increased, post-treatment minus pre-treatment > 30 s; decreased, post-treatment minus pre-treatment < −30 s; almost no altered; −30 s < post-treatment minus pre-treatment < 30 s) (*n* = 6 per group). (**D**) Comparison of differences in frequency and duration of following, sniffing, licking, and stress tail relevant to SHED-treated or untreated *SHANK3* mutant dogs before treatment and in 1-month post-treatment. The SHED-treated mutant dogs had a significantly decreasing duration of stress tail compared with the untreated mutant dogs (n = 3 per group). (**E**) Comparison of differences in frequency and duration of following, sniffing, licking, and stress tail relevant to SHED-treated or untreated *SHANK3* mutant dogs before treatment and in 3-month post-treatment. The SHED-treated mutant dogs had a significantly decreasing duration of stress tail compared with the untreated mutant dogs (*n* = 3 per group, each one was tested twice and all data were used for analysis). Data are presented as mean ± SEM. *P < .05, **P < .01, ****P < .0001.

To assess the effect of SHED transplantation, we compared the effect on beagle dog behavior of SNP in 1-month and 3-month post-treatment by the 3-chamber test. Untreated *SHANK3* mutant dogs (*SHANK3* + saline group) showed no significant differences in the duration of interactions between the Stranger1 and Stranger2, indicating impaired SNP in *SHANK3* mutant beagle dogs compared with age-matched wildtype dogs. Notably, SHED transplantation could restore normal SNP in the 1-month visit and maintain the long-term (3-month visit) improvement in the behavior of *SHANK3* mutant dogs (Fig. 2A). Comparison before and after SHED treatment of mutant dogs, the SNP was also significantly improved both in 1-month and 3-month visits (Fig. 2B). In addition, SHED-treated mutant dogs showed a significantly increased duration of interactions with the novel dogs (Stranger2) compared with pre-treatment, especially in the 1-month visit (Fig. 2B). Then, individual SNP of each mutant dog before treatment, in 1-month and 3-month visits was also evaluated by social time-difference (ST_dif_) between the Stranger2 and Stranger1. The heatmap was generated with green representing the positive ST_dif_ (Stranger2 > Stranger1) and red representing the negative ST_dif_ (Stranger2 < Stranger1) (Fig. 2C). In 1-month and 3-month visits, 5 of 6 SHED-treated mutant dogs showed increased ST_dif_ (post-treatment minus pre-treatment > 30 s); Only one SHED-treated mutant dog showed no altered ST_dif_ (−30 s < post-treatment minus pre-treatment < 30 s) (Fig. 2C). However, in the 1-month visit, 3 out of 6 untreated mutant dogs presented almost no altered ST_dif_, 2 mutant dogs exhibited increased ST_dif_, and one mutant dog showed decreased ST_dif_ (post-treatment minus pre-treatment < −30 s). In 3-month post-treatment, increased, decreased, and almost no altered ST_dif_ were noted in 2 untreated mutant dogs (Fig. 2C). The above results indicated the improvement of abnormal social novelty in SHED-treated animals.

To better assess the therapeutic effect of SHED transplantation, we performed a dog-and-experimenter interaction test, which cannot be adequately performed in mouse models. The SHED-treated mutant dogs had a significantly decreasing duration of stress tail compared with the untreated mutant dogs (Fig. 2D and E). Although not statistically significant, there was an improvement to different degrees for SHED-treated mutant dogs on both frequency and duration of following, sniffing, and licking in the 1-month visit (Fig. 2D). And in the 3-month visit, an increasing trend in the duration of sniffing and licking was also observed in the SHED-treated mutant dogs, but this did not reach statistical significance (Fig. 2E). In summary, social stress in *SHANK3* mutant dogs could be effectively alleviated by SHED treatment.

Taken together, these findings demonstrated that ASD-related abnormalities of SNP and social stress in *SHANK3* mutant dogs could be effectively alleviated by multiple transplantations of SHED.

### Stem Cells from Human Exfoliated Deciduous Teeth Transplantation Alters Serum Cytokine Levels

**Figure 3.**
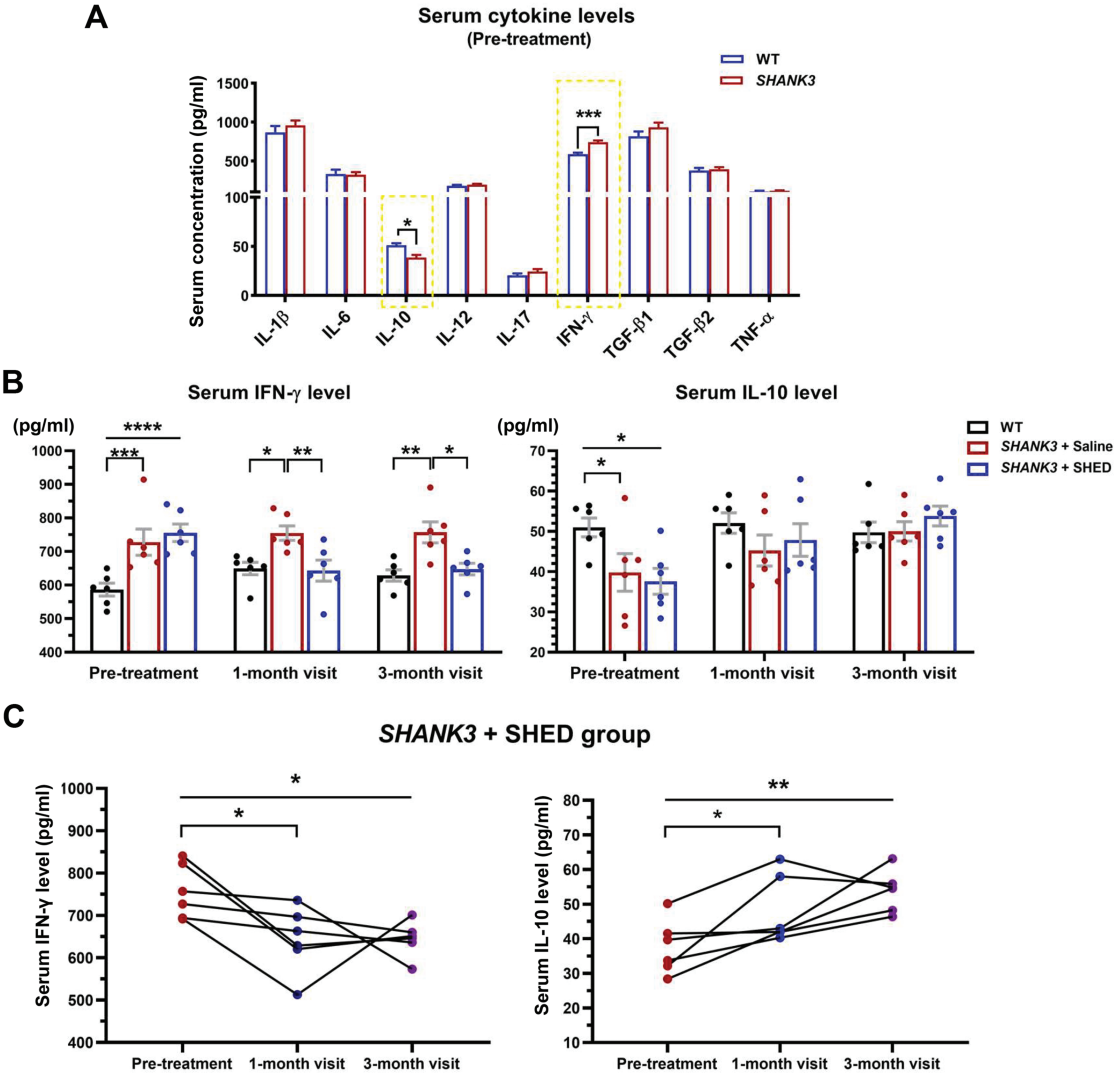
Effects of SHED transplantation on serum cytokine levels. (**A**) Comparison of IL-1β, IL-6, IL-10, IL-12, IL-17 IFN-γ, TGF-β1, TGF-β2, and TNF-α levels between *SHANK3* mutant and WT beagle dogs using canine cytokine ELISA kits. Significant differences were noted in the levels of serum IFN-γ and IL-10, which were indicated by yellow-dotted frames (*n* = 6 in WT, *n* = 12 in *SHANK3*). (**B**) Comparison of IFN-γ and IL-10 levels between WT and *SHANK3* mutant dogs before and after treatment in different groups (*n* = 6 per group). (**C**) Changes of IFN-γ and IL-10 levels in SHED-treated mutant dogs before and after treatment. Data are presented as mean ± SEM (*n* = 6). *P < .05, **P < .01, ***P < .001, ****P < .0001.

We observed a significantly increased level of serum interferon-γ (IFN-γ) and a significantly decreased level of serum interleukin-10 (IL-10) in *SHANK3* mutant beagle dogs compared with wild-type beagle dogs (Fig. 3A). After SHED transplantation, the level of serum IFN-γ was significantly reduced in comparison to that of untreated *SHANK3* mutant beagle dogs in the 1-month and 3-month visits (Fig. 3B). Compared with pre-treatment, SHED-treated mutant beagle dogs showed a decreasing trend of IFN-γ in 1-month post-treatment and an increasing trend of IL-10 in 3-month post-treatment (Fig. 3C). Also, SHED transplantation failed to significantly alter the levels of serum IL-1β, IL-6, IL-12, IL-17, TGF-β1, TGF-β2, and TNF-α in 1-month post-transplantation (Supplementary Fig. S2).

**Figure 4.**
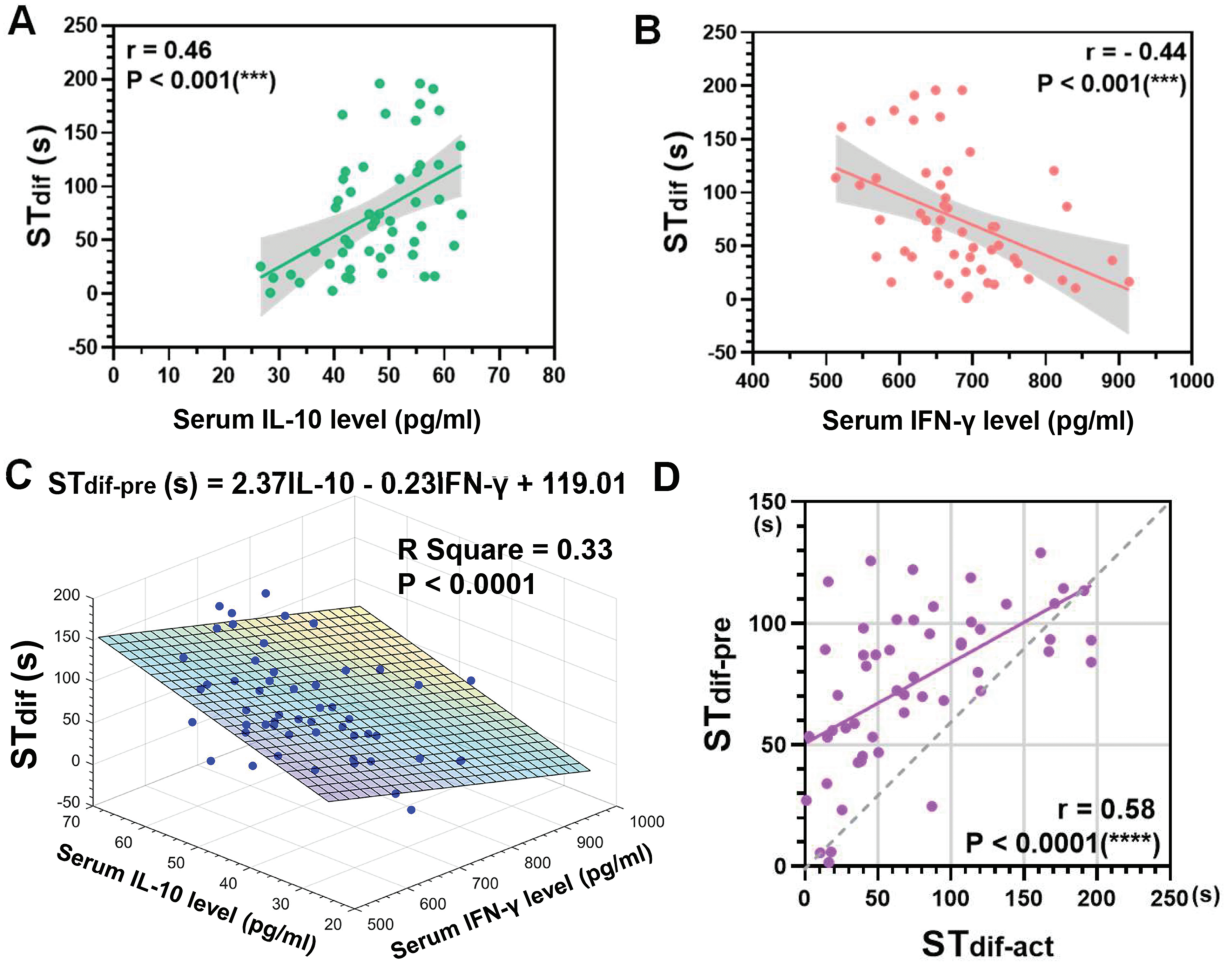
Correlation and regression analysis of ST_dif_ values and levels of serum IFN-γ and IL-10 in all *SHANK3* mutant dogs and WT controls. (**A**) Correlation analysis between ST_dif_ and the level of serum IL-10. Green dots represented the values of ST_dif_ calculated by social time-difference between the Stranger2 and Stranger1 with their corresponding IL-10 level for all *SHANK3* mutant dogs and WT controls. (Pearson’s *r* = .46, *P* < .001, *n* = 53 total dots, one datum was excluded as an obvious outlier). (**B**) Correlation analysis between ST_dif_ and the level of serum IFN-γ. Pink dots represented the values of ST_dif_ with their corresponding IFN-γ level for all *SHANK3* mutant dogs and WT controls. (Pearson’s *r* = −0.44, *P* < .001, *n* = 53 total dots, one datum was excluded as an obvious outlier). (**C**) Three-dimensional scatter diagram showing the actual values of ST_dif_ (ST_dif-act_) with their corresponding the levels of serum IFN-γ and IL-10 for all *SHANK3* mutant dogs and WT controls (*n* = 53 in total blue dots), and multiple linear regression model for predicting ST_dif_ (ST_dif-pre_) fitted by 2 independent variables of IFN-γ and IL-10 levels (color plane, *R*^2^ = 0.33, *P* < .0001). (**D**) Linear correlation between ST_dif-act_ and ST_dif-pre_. Dotted line represents perfect correlation (*r* = 1). Solid line represents actual correlation between the ST_dif-act_ and ST_dif-pre_ (*r* = .58, *P* < .0001). ***P < .001, ****P < .0001.

To evaluate the correlation between the levels of serum IFN-γ, IL10, and SNP, we used correlation analysis to show that IL-10 level was positively correlated with the degree of SNP (Pearson’s *r* = .46, *P* < .001, *n* = 53; Fig. 4A). Whereas IFN-γ level was negatively correlated with the degree of SNP (Pearson’s *r* = −0.44, *P* < .001, *n* = 53) (Fig. 4B). Based on the results above, multiple linear regression between IFN-γ, IL-10, and ST_dif_ in beagle dogs was constructed and the model for predicting ST_dif_ (ST_dif-pre_):

ST_dif-pre_ (s) = 2.37 × IL-10 (pg/mL)—0.23 × IFN-γ (pg/mL) **+** 119.01 (*R*^2^ = 0.33; *P* < .0001) (Fig. 4C).

To test the validity of the predicting model, we calculated the correlation between actual ST_dif_ (ST_dif-act_) and ST_dif-pre_ (Pearson’s *r* = .58, *P* < .0001, *n* = 53) (Fig. 4D).

## Discussion

Stem cells from human exfoliated deciduous teeth are derived from exfoliated deciduous teeth of children in the mixed dentition stage; they are a population of postnatal stem cells with the ability to differentiate into various cell types.^11^ Stem cells from human exfoliated deciduous teeth offer attractive advantages over other sources of postnatal stem cells, as they are derived from a source that is easy to harvest through non-invasive surgical procedures, are naturally disposed of, and present few ethical or legal concerns.^26^ Previous studies have demonstrated that SHED are highly proliferative cells compared to bone marrow MSCs and dental pulp MSCs.^27^ Since SHED derive from neural crest mesenchyme, they express a variety of embryonic stem cell markers, neural cell markers, stage-specific embryonic antigens, tumor recognition antigens and thus have enhanced potential to treat neural injuries or diseases.^11,27,28^ Our results indicated that SHED transplantation could effectively improve SNP and reduce social stress in *SHANK3* mutant dogs which exhibited several behaviors relevant to the core features of ASD.

To date, a few pre-clinical studies have demonstrated the therapeutic potential of stem cell transplantation in ASD animal models.^29,30^A widely studied ASD animal model is mouse models including the BTBR inbred mouse and the *SHANK3* mutant mouse, which have been used extensively in ASD research.^20,31-34^ However, species-dependent differences in brain structures and variable behavioral models make it difficult to use therapeutic information from ASD mice to design potential clinical trials. In recent years, gene-editing non-human primate animal models have been generated and demonstrated the value of ASD research.^17,18,35^ Although the application of non-human primate models has a promising future, the limitations at this stage exist, as follows. Cloning techniques could not facilitate the generation of monkeys with the same genetic modification and the rate of newborn mutant monkeys has been lower than expected.^36^ Therefore, due to ethical concerns, sample numbers, and economic issues, experiments of stem cell therapy for ASD with non-human primates have their limitations. Core features of behavioral phenotypes of ASD presented by heterozygous *SHANK3* mutant beagle dogs and the stable genetic background of these dogs have advantages for ASD research.

Deficits in SNP are a core symptom of ASD and tested by a 3-chamber test.^37^ In this study, SHED-treated mutant beagle dogs represented a significant improvement in SNP. The detailed mechanisms underlying the results need to be further explored and long-term maintenance of stem cell therapeutic effect needs to be also addressed. Moreover, the duration and frequency of following, sniffing, and licking as well as stress tail served as the main indexes of social interaction evaluated by the dog-and-experimenter interaction test. Interestingly, SHED transplantation significantly alleviated the social stress that mutant dogs experienced during social interaction with the human experimenter. Previous studies provided emerging evidence that children with ASD showed enhanced and sustained social stress that increases with aging.^38^ Social stress may also play a major role in the lives of adults with ASD and there is an inverse relationship between stress and social functioning.^39^ In addition, SHED-treated mutant dogs displayed a trend toward increased duration and frequency of following, sniffing, and licking behaviors, especially in 1-month visit, but there were no statistically significant differences. It is necessary to conduct additional experiments with the increased animal number to further examine the therapeutic effects of SHED in the future.

Several clinical trials using various stem cells to treat children with ASD have been conducted and some positive results have been obtained.^40-46^ Our results comply with these studies. In addition, we assessed the therapeutic potential of SHED in the *SHANK3* mutant beagle dogs. Although the mechanisms by which stem cells benefit ASD children have not been fully elucidated, it has been suggested that a large number of cytokines and extracellular vesicles produced and secreted by transplanted MSCs may regulate recipient immune responses.^37,47,48^ Transplanted MSCs can be trapped in the lung and undergo extensive apoptosis at 24 h post-transplantation.^49-51^ It has been suggested that apoptotic MSCs and their apoptotic vesicles can mediate tissue regeneration and immune regulation.^49-54^ Although the detailed etiology of ASD is unknown, it has been demonstrated that inflammation in the central nervous system and immune system disorders are important contributors to the pathogenesis of ASD.^55-58^*SHANK3* knockout mice showed elevated *Escherichia coli* lipopolysaccharide level in the liver and increased IL-6 level in the brain.^59^ It is reported that neural or systemic inflammatory stimuli can decrease *SHANK3* expression in the brain *via* NF-κB-related signaling regulation.^5,60^ Moreover, a considerable number of patients with Phelan-McDermid syndrome, caused by *SHANK3* mutation, show gastrointestinal symptoms and immune dysfunctions.^61^ These changes of molecular and cellular levels underlying ASD provide the basis for the therapeutic application of stem cells.

Our results indicated that *SHANK3* mutant beagle dogs showed an increased level of serum IFN-γ and a decreased level of serum IL-10 compared to wild-type dogs. SHED treatment reduced the serum IFN-γ level with a negative correlation of ST_dif_. IFN-γ have been proved to play an important role in T helper type 1 polarization and promote the cellular immune responses involving natural killer cells and CD8^+^ T cells, ultimately leading to the clearance of pathogens, cell debris, and tumor cells.^62^ Children with ASD showed a significant increase in immunostaining for IFN-γ in CD4^+^ T cells as well as in the mRNA and protein levels.^63^ A meta-analysis also provided evidence for higher concentrations of serum IFN-γ level in autistic patients compared with controls.^64^ Moreover, an increased mRNA level of IFN-γ was noted in the human brain of patients with ASD.^65^ Some mouse model studies confirmed upregulation of blood IFN-γ with autistic behaviors.^66,67^ Therefore, it seems that increased serum IFN-γ may serve as a biomarker reflecting neurological damage. However, the correlation between the levels of IFN-γ in the peripheral and central nervous systems and their relationship with autistic behaviors need to be further studied. On the other hand, MSC activation is dependent on the magnitude of IFN-γ exposure and IFN-γ stimulation-induced indoleamine 2,3-dioxygenase (IDO) expression, which is a major protein that mediates the immunosuppression of MSCs through the IFN-γ-Janus kinase (JAK)-signal transducer and activator of transcription (STAT) 1 pathway.^68-70^ In-depth proteomic analysis of IFN-γ-treated bone marrow MSCs indicated that approximately 30% of alterations in the expression of proteins were associated with immune regulation, particularly immune suppression.^71^ SHED show enhanced immune-modulatory properties, probably correlated with increased HLA-G expression in response to IFN-γ treatment.^72^ The increased level of peripheral IFN-γ in *SHANK3* heterozygous beagle dogs may provide natural IFN-γ preconditioning for SHED.

After SHED treatment, an increased level of serum IL-10 and a positive correlation between the IL-10 level and ST_dif_ were revealed. IL-10 is an important immune regulator which inhibits undesirable innate and acquired immunity.^73^ It can be released by various cell types including B lymphocytes, monocytes/macrophages, T helper cell 2, and T regulatory cells.^73,74^ Previous studies indicated the low level of anti-inflammatory molecular IL-10 was associated with autism-related behavioral impairments and children with ASD showed a significantly elevated IFN-γ/IL-10 ratio when compared to the control group.^75-77^ Altered levels of serum cytokines reflect a potential participation of the immune responses in the ASD development.

The ST_dif_ of beagle dogs was significantly correlated with a decreased level of serum IFN-γ and an increased level of IL-10. Previous studies showed that the ratio of IFN-γ:IL-10 is considered as a meaningful marker to define the disease severity in pulmonary or coronary heart disease and to identify the high-risk individuals from unaffected sibs of patients with nonsegmental vitiligo.^78-80^ Here, we identified a model fitted by levels of serum IFN-γ and IL-10 to evaluate ST_dif_, indicating the degree of autistic severity and therapeutic outcomes of stem cells. In the future, we need to further explore the below issues. Firstly, extended follow-up and large-scale study of *SHANK3* mutant beagle dogs with SHED transplantation will be required to further optimize therapeutic effects and explore therapeutic mechanisms in depth. Secondly, the regression model is based on serum cytokine measurements, which can be easily affected by both biological and technical factors. Although these cytokine concentrations from the same individual remained stable on repeat measurements, we cannot fully exclude the effect of unmeasured confounders. Thirdly, the *R*^2^ value of our model was not ideal, but it is acceptable to consider the complex nature of ASD.

## Summary

Our study demonstrates that intravenous SHED transplantation can effectively alleviate the autistic-like symptoms of impaired SNP and obvious social stress in *SHANK3* mutant beagle dogs. These SNP improvements are accompanied by an increase in the level of serum IL-10 and a decrease in the level of IFN-γ. Moreover, a linear regression model for ST_dif_ prediction is constructed fitted by levels of serum IFN-γ and IL-10 to evaluate the degree of autistic severity and therapeutic outcomes. Overall, our study provides the first evidence that the *SHANK3* mutant beagle dog model may be valuable for evaluating SHED-mediated therapy in ASD. Stem cells from human exfoliated deciduous teeth transplantation may serve as a simple, safe, and effective therapy for ASD.

## Supplementary Material

szac028_suppl_Supplementary_Figures

## Data Availability

The data that support the findings in this study are available from the corresponding author upon reasonable request.

## References

[1] Lord C , Elsabbagh M, Baird G, et al. Autism spectrum disorder. Lancet. 2018;392(10146):508-520. 30078460 10.1016/S0140-6736(18)31129-2 PMC7398158

[2] Lai MC , Lombardo MV, Baron-Cohen S. Autism. Lancet. 2014;383(9920):896-910. 24074734 10.1016/S0140-6736(13)61539-1

[3] Maenner MJ , Shaw KA, Bak J, et al. Prevalence of autism spectrum disorder among children aged 8 years—autism and developmental disabilities monitoring network, 11 sites, United States, 2016. MMWR Surveill Summ. 2020;69(4):1. 10.15585/mmwr.ss6904a1 PMC7119644 32214087

[4] Lord C , Brugha TS, Charman T, et al. Autism spectrum disorder. Nat Rev Dis Primers. 2020;6(1):5. 31949163 10.1038/s41572-019-0138-4 PMC8900942

[5] Bai D , Yip BHK, Windham GC, et al. Association of genetic and environmental factors with autism in a 5-country cohort. JAMA Psychiatry. 2019;76(10):1035-1043. 31314057 10.1001/jamapsychiatry.2019.1411 PMC6646998

[6] Iakoucheva LM , Muotri AR, Sebat J. Getting to the cores of autism. Cell. 2019;178(6):1287-1298. 31491383 10.1016/j.cell.2019.07.037 PMC7039308

[7] Bralten J , van Hulzen KJ, Martens MB, et al. Autism spectrum disorders and autistic traits share genetics and biology. Mol Psychiatry. 2018;23(5):1205-1212. 28507316 10.1038/mp.2017.98 PMC5984081

[8] Kim JY , Son MJ, Son CY, et al. Environmental risk factors and biomarkers for autism spectrum disorder: an umbrella review of the evidence. Lancet Psychiatry. 2019;6(7):590-600. 31230684 10.1016/S2215-0366(19)30181-6

[9] Siniscalco D , Antonucci N. Cellular therapy for autism spectrum disorder: a step forward to the optimal treatments. Ann Transl Med. 2019;7(Suppl 3):S110. 31576317 10.21037/atm.2019.05.12 PMC6685839

[10] Carpenter KLH , Major S, Tallman C, et al. White matter tract changes associated with clinical improvement in an open-label trial assessing autologous umbilical cord blood for treatment of young children with autism. Stem Cells Transl Med. 2019;8(2):138-147. 30620122 10.1002/sctm.18-0251 PMC6344899

[11] Miura M , Gronthos S, Zhao M, et al. SHED: stem cells from human exfoliated deciduous teeth. Proc Natl Acad Sci USA. 2003;100(10):5807-5812. 12716973 10.1073/pnas.0937635100 PMC156282

[12] Taghipour Z , Karbalaie K, Kiani A, et al. Transplantation of undifferentiated and induced human exfoliated deciduous teeth-derived stem cells promote functional recovery of rat spinal cord contusion injury model. Stem Cells Dev. 2012;21(10):1794-1802. 21970342 10.1089/scd.2011.0408

[13] Nicola F , Marques MR, Odorcyk F, et al. Stem cells from human exfoliated deciduous teeth modulate early astrocyte response after spinal cord contusion. Mol Neurobiol. 2019;56(1):748-760. 29796991 10.1007/s12035-018-1127-4

[14] Martens W , Bronckaers A, Politis C, et al. Dental stem cells and their promising role in neural regeneration: an update. Clin Oral Investig. 2013;17(9):1969-1983. 10.1007/s00784-013-1030-3 23846214

[15] Yamagata M , Yamamoto A, Kako E, et al. Human dental pulp-derived stem cells protect against hypoxic-ischemic brain injury in neonatal mice. Stroke. 2013;44(2):551-554. 23238858 10.1161/STROKEAHA.112.676759

[16] Shimojima C , Takeuchi H, Jin S, et al. Conditioned medium from the stem cells of human exfoliated deciduous teeth ameliorates experimental autoimmune encephalomyelitis. J Immunol. 2016;196(10):4164-4171. 27053763 10.4049/jimmunol.1501457

[17] Tu Z , Zhao H, Li B, et al. CRISPR/Cas9-mediated disruption of SHANK3 in monkey leads to drug-treatable autism-like symptoms. Hum Mol Genet. 2019;28(4):561-571. 30329048 10.1093/hmg/ddy367 PMC6489410

[18] Zhou Y , Sharma J, Ke Q, et al. Atypical behaviour and connectivity in SHANK3-mutant macaques. Nature. 2019;570(7761):326-331. 31189958 10.1038/s41586-019-1278-0

[19] Monteiro P , Feng GP. SHANK proteins: roles at the synapse and in autism spectrum disorder. Nat Rev Neurosci. 2017;18(3):147-157. 28179641 10.1038/nrn.2016.183

[20] Jiang YH , Ehlers MD. Modeling autism by SHANK gene mutations in mice. Neuron. 2013;78(1):8-27. 23583105 10.1016/j.neuron.2013.03.016 PMC3659167

[21] Gronthos S , Mankani M, Brahim J, et al. Postnatal human dental pulp stem cells (DPSCs) in vitro and in vivo. Proc Natl Acad Sci USA. 2000;97(25):13625-13630. 11087820 10.1073/pnas.240309797 PMC17626

[22] Moy SS , Nadler JJ, Perez A, et al. Sociability and preference for social novelty in five inbred strains: an approach to assess autistic-like behavior in mice. Genes Brain Behav. 2004;3(5):287-302. 15344922 10.1111/j.1601-1848.2004.00076.x

[23] Silverman JL , Yang M, Lord C, et al. Behavioural phenotyping assays for mouse models of autism. Nat Rev Neurosci. 2010;11(7):490-502. 20559336 10.1038/nrn2851 PMC3087436

[24] Siwak CT , Tapp PD, Milgram NW. Effect of age and level of cognitive function on spontaneous and exploratory behaviors in the beagle dog. Learn Memory. 2001;8(6):317-325. 10.1101/lm.41701 PMC311391 11773431

[25] Siniscalchi M , d’Ingeo S, Minunno M, et al. Communication in dogs. Animals. 2018;8(8):131. 10.3390/ani8080131 PMC6116041 30065156

[26] Huang GT , Gronthos S, Shi S. Mesenchymal stem cells derived from dental tissues vs. those from other sources: their biology and role in regenerative medicine. J Dent Res. 2009;88(9):792-806. 19767575 10.1177/0022034509340867 PMC2830488

[27] Isobe Y , Koyama N, Nakao K, et al. Comparison of human mesenchymal stem cells derived from bone marrow, synovial fluid, adult dental pulp, and exfoliated deciduous tooth pulp. Int J Oral Maxillofac Surg. 2016;45(1):124-131. 26235629 10.1016/j.ijom.2015.06.022

[28] Kerkis I , Kerkis A, Dozortsev D, et al. Isolation and characterization of a population of immature dental pulp stem cells expressing OCT-4 and other embryonic stem cell markers. Cells Tissues Organs. 2006;184(3-4):105-116. 17409736 10.1159/000099617

[29] Perets N , Segal-Gavish H, Gothelf Y, et al. Long term beneficial effect of neurotrophic factors-secreting mesenchymal stem cells transplantation in the BTBR mouse model of autism. Behav Brain Res. 2017;331:254-260. 28392323 10.1016/j.bbr.2017.03.047

[30] Segal-Gavish H , Karvat G, Barak N, et al. Mesenchymal stem cell transplantation promotes neurogenesis and ameliorates autism related behaviors in BTBR mice. Autism Res. 2016;9(1):17-32. 26257137 10.1002/aur.1530

[31] Faraji J , Karimi M, Lawrence C, et al. Non-diagnostic symptoms in a mouse model of autism in relation to neuroanatomy: the BTBR strain reinvestigated. Transl Psychiatry. 2018;8(1):234. 30367028 10.1038/s41398-018-0280-x PMC6203744

[32] Coretti L , Cristiano C, Florio E, et al. Sex-related alterations of gut microbiota composition in the BTBR mouse model of autism spectrum disorder. Sci Rep. 2017;7:45356. 28349974 10.1038/srep45356 PMC5368984

[33] Uddin MN , Yao Y, Mondal T, et al. Immunity and autoantibodies of a mouse strain with autistic-like behavior. Brain Behav Immun Health. 2020;4:100069. 34589851 10.1016/j.bbih.2020.100069 PMC8474232

[34] Mizuno S , Hirota JN, Ishii C, et al. Comprehensive profiling of gene expression in the cerebral cortex and striatum of BTBRTF/ArtRbrc mice compared to C57BL/6J mice. Front Cell Neurosci. 2020;14:595607. 33362469 10.3389/fncel.2020.595607 PMC7758463

[35] Liu Z , Li X, Zhang JT, et al. Autism-like behaviours and germline transmission in transgenic monkeys overexpressing MeCP2. Nature. 2016;530(7588):98-102. 26808898 10.1038/nature16533

[36] Tardif SD , Coleman K, Hobbs TR, et al. IACUC review of nonhuman primate research. ILAR J. 2013;54(2):234-245. 24174445 10.1093/ilar/ilt040 PMC3814393

[37] Siniscalco D , Kannan S, Semprun-Hernandez N, et al. Stem cell therapy in autism: recent insights. Stem Cells Cloning. 2018;11:55-67. 30425534 10.2147/SCCAA.S155410 PMC6204871

[38] Corbett BA , Schupp CW, Lanni KE. Comparing biobehavioral profiles across 2 social stress paradigms in children with and without autism spectrum disorders. Mol Autism. 2012;3(1):13. 23158965 10.1186/2040-2392-3-13 PMC3533919

[39] Bishop-Fitzpatrick L , Mazefsky CA, Minshew NJ, et al. The relationship between stress and social functioning in adults with autism spectrum disorder and without intellectual disability. Autism Res. 2015;8(2):164-173. 25524571 10.1002/aur.1433 PMC4412754

[40] Chez M , Lepage C, Parise C, et al. Safety and observations from a placebo-controlled, crossover study to assess use of autologous umbilical cord blood stem cells to improve symptoms in children with autism. Stem Cell Transl Med. 2018;7(4):333-341. 10.1002/sctm.17-0042 PMC5866927 29405603

[41] Dawson G , Sun JM, Davlantis KS, et al. Autologous cord blood infusions are safe and feasible in young children with autism spectrum disorder: results of a single-center phase I open-label trial. Stem Cell Transl Med. 2017; 6(5): 1332-1339. 10.1002/sctm.16-0474 PMC5442708 28378499

[42] Sun JSM , Dawson G, Franz L, et al. Infusion of human umbilical cord tissue mesenchymal stromal cells in children with autism spectrum disorder. Stem Cell Transl Med. 2020;9(10):1137-1146. 10.1002/sctm.19-0434 PMC7519773 32531111

[43] Thanh LN , Nguyen HP, Ngo MD, et al. Outcomes of bone marrow mononuclear cell transplantation combined with interventional education for autism spectrum disorder. Stem Cell Transl Med. 2021;10(1):14-26. 10.1002/sctm.20-0102 PMC7780798 32902182

[44] Bradstreet JJ , Sych N, Antonucci N, et al. Efficacy of fetal stem cell transplantation in autism spectrum disorders: an open-labeled pilot study. Cell Transplant. 2014;23:S105-S112. 25302490 10.3727/096368914X684916

[45] Lv YT , Zhang Y, Liu M, et al. Transplantation of human cord blood mononuclear cells and umbilical cord-derived mesenchymal stem cells in autism. J Transl Med. 2013;11. 10.1186/1479-5876-11-196 PMC3765833 23978163

[46] Villarreal-Martinez L , Gonzalez-Martinez G, Saenz-Flores M, et al. Stem cell therapy in the treatment of patients with autism spectrum disorder: a systematic review and meta-analysis. Stem Cell Rev Rep. 2022;18(1):155-164. 34515938 10.1007/s12015-021-10257-0

[47] Siniscalco D , Bradstreet JJ, Sych N, et al. Perspectives on the use of stem cells for autism treatment. Stem Cells Int. 2013;2013:262438. 24222772 10.1155/2013/262438 PMC3810518

[48] Alessio N , Brigida AL, Peluso G, et al. Stem cell-derived exosomes in autism spectrum disorder. Int J Environ Res Public Health. 2020;17(3):944. 10.3390/ijerph17030944 PMC7037429 32033002

[49] Lee RH , Pulin AA, Seo MJ, et al. Intravenous hMSCs improve myocardial infarction in mice because cells embolized in lung are activated to secrete the anti- inflammatory protein TSG-6. Cell Stem Cell. 2009;5(1):54-63. 19570514 10.1016/j.stem.2009.05.003 PMC4154377

[50] Liu S , Jiang L, Li H, et al. Mesenchymal stem cells prevent hypertrophic scar formation via inflammatory regulation when undergoing apoptosis. J Invest Dermatol. 2014;134(10):2648-2657. 24714203 10.1038/jid.2014.169

[51] Fu Y , Sui B, Xiang L, et al. Emerging understanding of apoptosis in mediating mesenchymal stem cell therapy. Cell Death Dis. 2021;12(6):596. 34108448 10.1038/s41419-021-03883-6 PMC8190192

[52] Galleu A , Riffo-Vasquez Y, Trento C, et al. Apoptosis in mesenchymal stromal cells induces in vivo recipient-mediated immunomodulation. Sci Transl Med. 2017;9(416):eaam7828. 29141887 10.1126/scitranslmed.aam7828

[53] Liu D , Kou X, Chen C, et al. Circulating apoptotic bodies maintain mesenchymal stem cell homeostasis and ameliorate osteopenia via transferring multiple cellular factors. Cell Res. 2018;28(9):918-933. 30030518 10.1038/s41422-018-0070-2 PMC6123409

[54] Kou X , Xu X, Chen C, et al. The Fas/Fap-1/Cav-1 complex regulates IL-1RA secretion in mesenchymal stem cells to accelerate wound healing. Sci Transl Med. 2018;10(432):eaai8524. 29540618 10.1126/scitranslmed.aai8524 PMC6310133

[55] Vargas DL , Nascimbene C, Krishnan C, et al. Neuroglial activation and neuroinflammation in the brain of patients with autism. Ann Neurol. 2005;57(1):67-81. 15546155 10.1002/ana.20315

[56] Jyonouchi H , Geng L, Ruby A, et al. Dysregulated innate immune responses in young children with autism spectrum disorders: Their relationship to gastrointestinal symptoms and dietary intervention. Neuropsychobiology. 2005;51(2):77-85. 15741748 10.1159/000084164

[57] Jyonouchi H , Sun SN, Le H. Proinflammatory and regulatory cytokine production associated with innate and adaptive immune responses in children with autism spectrum disorders and developmental regression. J Neuroimmunol. 2001;120(1-2):170-179. 11694332 10.1016/s0165-5728(01)00421-0

[58] Zimmerman AW , Jyonouchi H, Comi AM, et al. Cerebrospinal fluid and serum markers of inflammation in autism. Pediatr Neurol. 2005;33(3):195-201. 16139734 10.1016/j.pediatrneurol.2005.03.014

[59] Sauer AK , Bockmann J, Steinestel K, et al. Altered intestinal morphology and microbiota composition in the autism spectrum disorders associated SHANK3 mouse model. Int J Mol Sci. 2019;20(9):2134. 10.3390/ijms20092134 PMC6540607 31052177

[60] Bey AL , Gorman MP, Gallentine W, et al. Subacute neuropsychiatric syndrome in girls with *SHANK3* mutations responds to immunomodulation. Pediatrics. 2020;145(2):e20191490. 32015180 10.1542/peds.2019-1490 PMC7802010

[61] Ricciardello A , Tomaiuolo P, Persico AM. Genotype-phenotype correlation in Phelan-McDermid syndrome: A comprehensive review of chromosome 22q13 deleted genes. Am J Med Genet A. 2021;185(7):2211-2233. 33949759 10.1002/ajmg.a.62222 PMC8251815

[62] Yanagawa Y , Iwabuchi K, Onoe K. Co-operative action of interleukin-10 and interferon-gamma to regulate dendritic cell functions. Immunology. 2009;127(3):345-353. 19191915 10.1111/j.1365-2567.2008.02986.x PMC2712103

[63] Ahmad SF , Nadeem A, Ansari MA, et al. Upregulation of IL-9 and JAK-STAT signaling pathway in children with autism. Prog Neuro-Psychoph. 2017;79:472-480. 10.1016/j.pnpbp.2017.08.002 28802860

[64] Saghazadeh A , Ataeinia B, Keynejad K, et al. A meta-analysis of pro-inflammatory cytokines in autism spectrum disorders: effects of age, gender, and latitude. J Psychiatr Res. 2019;115:90-102. 31125917 10.1016/j.jpsychires.2019.05.019

[65] Patel N , Crider A, Pandya CD, et al. Altered mRNA levels of glucocorticoid receptor, mineralocorticoid receptor, and co-chaperones (FKBP5 and PTGES3) in the middle frontal gyrus of autism spectrum disorder subjects. Mol Neurobiol. 2016;53(4):2090-2099. 25912394 10.1007/s12035-015-9178-2

[66] Zhang YB , Gao DH, Kluetzman K, et al. The maternal autoimmune environment affects the social behavior of offspring. J Neuroimmunol. 2013;258(1-2):51-60. 23537887 10.1016/j.jneuroim.2013.02.019

[67] Alfawaz HA , Bhat RS, Al-Ayadhi L, et al. Protective and restorative potency of Vitamin D on persistent biochemical autistic features induced in propionic acid-intoxicated rat pups. BMC Complem Altern Med. 2014;14:416. 10.1186/1472-6882-14-416 PMC4230722 25344727

[68] Li H , Liu Q, Gao X, et al. IFN-gamma gene loaded human umbilical mesenchymal stromal cells targeting therapy for Graft-versus-host disease. Int J Pharm. 2021;592:120058. 33220383 10.1016/j.ijpharm.2020.120058

[69] Kim DS , Jang IK, Lee MW, et al. Enhanced immunosuppressive properties of human mesenchymal stem cells primed by interferon-gamma. EBioMedicine 2018;28:261-273. 29366627 10.1016/j.ebiom.2018.01.002 PMC5898027

[70] Yu Y , Yoo SM, Park HH, et al. Preconditioning with interleukin-1 beta and interferon-gamma enhances the efficacy of human umbilical cord blood-derived mesenchymal stem cells-based therapy via enhancing prostaglandin E2 secretion and indoleamine 2,3-dioxygenase activity in dextran sulfate sodium-induced colitis. J Tissue Eng Regen Med. 2019;13(10):1792-1804. 31293088 10.1002/term.2930

[71] Klinker MW , Marklein RA, Lo Surdo JL, et al. Morphological features of IFN-gamma-stimulated mesenchymal stromal cells predict overall immunosuppressive capacity. Proc Natl Acad Sci USA. 2017;114(13):E2598-E2607. 28283659 10.1073/pnas.1617933114 PMC5380055

[72] Guan Q , Ezzati P, Spicer V, et al. Interferon gamma induced compositional changes in human bone marrow derived mesenchymal stem/stromal cells. Clin Proteomics. 2017;14:26. 28694743 10.1186/s12014-017-9161-1 PMC5501357

[73] Couper KN , Blount DG, Riley EM. IL-10: the master regulator of immunity to infection. J Immunol. 2008;180(9):5771-5777. 18424693 10.4049/jimmunol.180.9.5771

[74] Moore KW , de Waal Malefyt R, Coffman RL, et al. Interleukin-10 and the interleukin-10 receptor. Annu Rev Immunol. 2001;19:683-765. 11244051 10.1146/annurev.immunol.19.1.683

[75] Molloy CA , Morrow AL, Meinzen-Derr J, et al. Elevated cytokine levels in children with autism spectrum disorder. J Neuroimmunol. 2006;172(1-2):198-205. 16360218 10.1016/j.jneuroim.2005.11.007

[76] Ross HE , Guo Y, Coleman K, et al. Association of IL-12p70 and IL-6:IL-10 ratio with autism-related behaviors in 22q11.2 deletion syndrome: a preliminary report. Brain Behav Immunol. 2013;31:76-81. 10.1016/j.bbi.2012.12.021 PMC3669236 23353117

[77] Moaaz M , Youssry S, Elfatatry A, et al. Th17/Treg cells imbalance and their related cytokines (IL-17, IL-10 and TGF-beta) in children with autism spectrum disorder. J Neuroimmunol. 2019;337:577071. 31671361 10.1016/j.jneuroim.2019.577071

[78] Jamil B , Shahid F, Hasan Z, et al. Interferon gamma/IL10 ratio defines the disease severity in pulmonary and extra pulmonary tuberculosis. Tuberculosis (Edinb). 2007;87(4):279-287. 17532265 10.1016/j.tube.2007.03.004

[79] Ala Y , Pasha MK, Rao RN, et al. Association of IFN-gamma: IL-10 cytokine ratio with nonsegmental vitiligo pathogenesis. Autoimmune Dis. 2015;2015:423490. 26442157 10.1155/2015/423490 PMC4579304

[80] Liang K , Dong SR, Peng H. Serum levels and clinical significance of IFN-gamma and IL-10 in patients with coronary heart disease. Eur Rev Med Pharmacol Sci. 2016;20(7):1339-1343. 27097956

