# Supplementary material for: Stem Cells from Human Exfoliated Deciduous Teeth Ameliorate Autistic-Like Behaviors of SHANK3 Mutant Beagle Dogs
Source: Stem Cells Transl Med. 2022 May 24;11(7):778–89. doi: 10.1093/stcltm/szac028 (PMC9299510; doi:10.1093/stcltm/szac028)
Supplement: szac028_suppl_Supplementary_Figures [file szac028_suppl_supplementary_figures.pdf]

Supplemental Information

Stem cells from human exfoliated deciduous teeth ameliorate autistic-like behaviors of *SHANK3* mutant beagle dogs

Figure S1

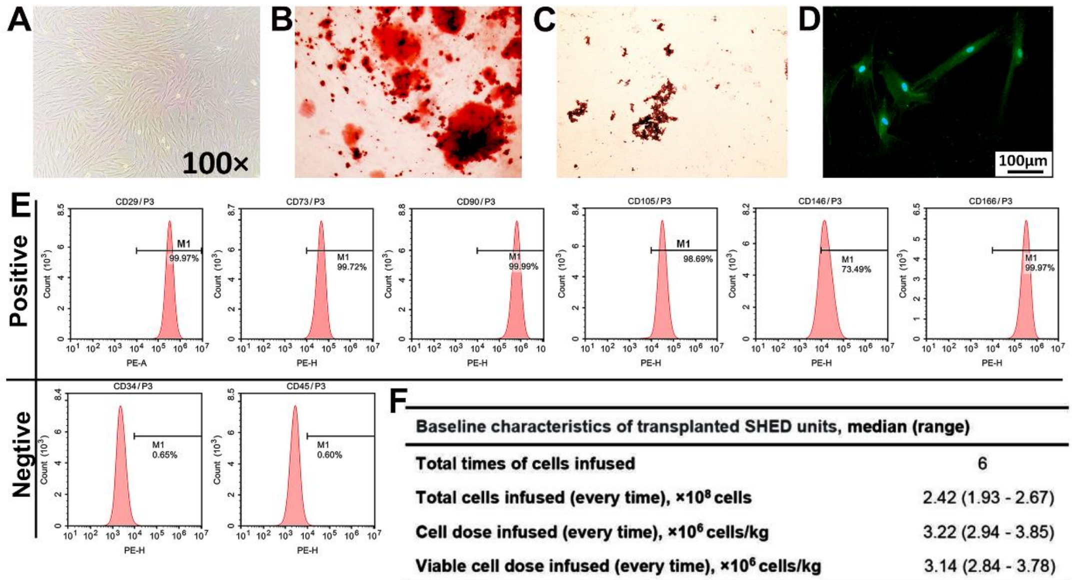

Baseline characteristics of transplanted SHED units, median (range)	
Total times of cells infused	6
Total cells infused (every time), ×10⁸ cells	2.42 (1.93 - 2.67)
Cell dose infused (every time), ×10⁶ cells/kg	3.22 (2.94 - 3.85)
Viable cell dose infused (every time), ×10⁶ cells/kg	3.14 (2.84 - 3.78)

FIGURE S1 Characterization of SHED. (A) Morphology of cultured SHED at passage 3. (B) Osteogenic differentiation of SHED. (C) Adipogenic differentiation of SHED. (D) Neurogenic differentiation of SHED. Green, Neun; Blue, DAPI. Bar, 100μm. (E) Flow cytometric analysis of SHED surface molecules. SHED were positive for mesenchymal lineage markers CD29, CD73, CD90, CD105, CD146, and CD166, but were negative for hematopoietic and endothelial markers CD34 and CD45. (F) Baseline characteristics of transplanted SHED.

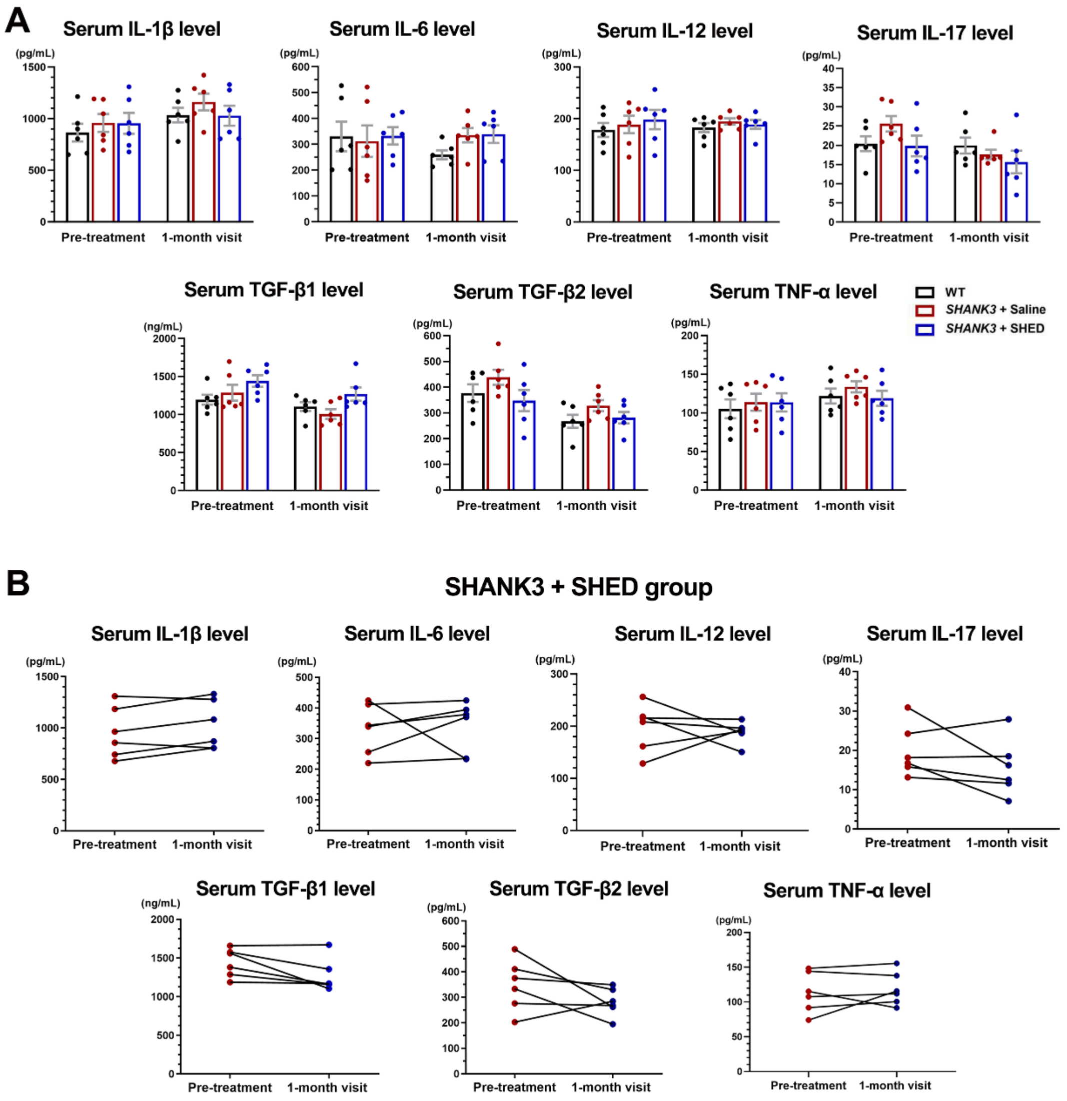

FIGURE S2. Effects of SHED transplantation on the levels of serum IL-1β, IL-6, IL-12, IL-17, TGF-β1, TGF-β2, and TNF-α. (A) The levels of serum IL-1β, IL-6, IL-12, IL-17, TGF-β1, TGF-β2, and TNF-α between WT and *SHANK3* mutant dogs before and after treatment with SHED (n = 6 per group). (B) The levels of serum IL-1β, IL-6, IL-12, IL-17, TGF-β1, TGF-β2, and TNF-α in SHED-treated mutant dogs. Data are presented as mean ± SEM (n = 6).
